# From one to the other: responding to Ebola cases on either side of the line

**DOI:** 10.11604/pamj.supp.2017.27.1.12568

**Published:** 2017-05-28

**Authors:** Rebecca Day Merrill, Sadie Elizabeth Ward, Marydale Cauble Oppert, Dana Allison Schneider

**Affiliations:** 1 International Border Team, Division of Global Migration and Quarantine, U.S. Centers for Disease Control and Prevention (CDC), Atlanta, USA

**Keywords:** Public Health, Epidemiology, Ebola virus disease, Sierra Leone

## Abstract

This case study is adapted from events that occurred along the Sierra Leone and Guinea land border during the 2014-2016 Ebola epidemic in West Africa. The response activities involved Sierra Leone and Guinea officials, along with assistance from U.S. Centers for Disease Control and Prevention (CDC) and the World Health Organisation (WHO). This case study builds upon an understanding of basic surveillance systems and outbreak response activities. Through this exercise, students will understand how to incorporate communication and coordination into surveillance and response efforts with counterparts across the border in neighbouring countries. This integration is important to reduce the spread of communicable diseases between neighbouring countries. The time required to complete this case study is 2-3 hours.

## How to use this case study

**General instructions:** this case study in applied epidemiology allows students to practise skills in the classroom setting to address real-world public health problems. It is suited to reinforcing principles and skills already covered in a lecture or background reading. Ideally, one or two instructors facilitate the case study in a classroom or conference room for up to 20 students. Traditionally, the instructor directs a participant to read aloud a paragraph or two, going around the room and giving each participant a chance to read. When a participant reads a question, the instructor guides all participants’ responses by engaging in a discussion and using diagrams, when relevant. Sometimes, the instructor will split the class into groups to complete activities or to assume different sides of the discussion when answering a question. Through these teaching methods, participants learn from each other, not just from the instructors.

**Audience:** residents in Field Epidemiology Training Programs (FETPs), Field Epidemiology and Laboratory Training Programs (FELTPs), and other health professionals who are interested in this topic.

**Prerequisites:** participants should have received lectures or other instructions in general public health surveillance and outbreak response.

**Materials needed:** flip chart or whiteboard, markers

**Level of training and associated public health activity:** novice - surveillance, public health preparedness and response

**Time required:** 2-3 hours

**Language:** English

## Case study material

- Download the case study student guide (PDF - 1.38 MB)
- Request the case study facilitator guide
